# Apigenin for Depression and Anxiety: A Systematic Review of Preclinical Studies

**DOI:** 10.5812/ijpr-167153

**Published:** 2026-05-19

**Authors:** Jin Wang, Kun Shi

**Affiliations:** 1 Department of Geriatric, The First People's Hospital of Lanzhou, Lanzhou, China; 2 Department of Psychiatry and Psychology, Lintong Rehabilitation and Convalescent Center, Xi'an, China

**Keywords:** Apigenin, Depression, Anxiety, Antioxidant, Systematic Review

## Abstract

**Context:**

Depression and anxiety are debilitating disorders with complex pathophysiologies associated with neurotransmitter deficits, neuroinflammation, and oxidative stress. Apigenin, a dietary flavonoid, has therapeutic potential because of its neuroprotective properties.

**Evidence Acquisition:**

We assessed the antidepressant and anxiolytic effects of apigenin in rodent models, focusing on behavioral outcomes, mechanisms involving neuroinflammation, oxidative stress, and neurotransmitter balance, and relevant molecular pathways. A systematic search of PubMed, Scopus, and Embase was conducted through July 2025. In vivo rodent studies evaluating the effects of apigenin on depression or anxiety were included. Exclusion criteria comprised studies addressing other disorders, apigenin derivatives, or non-original research. Data on study design, model, treatment, outcomes, and mechanisms were extracted.

**Results:**

Of 953 screened records, 21 studies met the inclusion criteria. Preclinical evidence consistently indicated that apigenin ameliorated depressive- and anxiety-like behaviors across diverse rodent models, frequently demonstrating effects comparable to or greater than those of conventional pharmacological treatments. Mechanistically, apigenin mediated its neuropsychological effects through multiple pathways, including reducing oxidative and nitrosative stress, inhibiting neuroinflammation, modulating monoaminergic neurotransmission, upregulating neurotrophic factors such as brain-derived neurotrophic factor (BDNF) and cAMP response element-binding protein (CREB), and regulating energy metabolism and neurogenesis. Apigenin exhibited a favorable safety profile across all included studies, even at high doses.

**Conclusions:**

In rodent models, apigenin exhibits substantial antidepressant and anxiolytic effects through complex mechanisms involving redox modulation, anti-inflammatory activity, neurotransmitter regulation, and neurotrophic support. These findings indicate that apigenin warrants further translational investigation and potential therapeutic development for treating depression and anxiety disorders.

## 1. Context

Depression is a chronic, complex psychiatric disorder characterized by a constellation of symptoms, including mood disturbances, diminished capacity to experience pleasure (anhedonia), and persistent fatigue (1). Recognized as a leading cause of disability worldwide (2, 3), the pathophysiology of depression is thought to involve reduced levels of critical neurotransmitters, notably norepinephrine and serotonin, at neuronal synapses, ultimately impairing mood regulation (4). Beyond neurotransmitter deficits, accumulating evidence underscores the central role of inflammatory processes, as demonstrated by elevated concentrations of pro-inflammatory cytokines, such as IL-6 and TNF-α, in both the brains and peripheral tissues of individuals with depression (4, 5). Furthermore, dysregulation of the hypothalamic-pituitary-adrenal (HPA) axis, reduced antioxidant defenses, and increased oxidative stress have been implicated in depressive pathology (6, 7, 8).

Anxiety disorders are also neuropsychiatric conditions that substantially affect psychological and physical well-being. These disorders are characterized by impaired emotional stability, heightened stress reactivity, and reduced quality of life (9). A complex interplay of social, environmental, and physiological factors can disrupt bodily homeostasis, affect neural signaling, and predispose individuals to a spectrum of anxiety-related health issues. These issues range from post-traumatic stress and clinical depression to chronic pain, exacerbation of systemic diseases such as asthma and diabetes, increased susceptibility to inflammatory states, and various phobias (10, 11). The manifestation of anxiety varies widely among individuals; however, typical symptoms include restlessness, chest pain, a sense of impending danger, excessive sweating, chills, numbness, hot flashes, and shortness of breath (12). Moreover, individuals with chronic health conditions, certain dietary patterns such as excessive caffeine intake, or specific life experiences are at heightened risk of developing anxiety disorders (12, 13).

Cognitive decline, often characterized by impaired working memory, learning and attention deficits, and reduced executive function, is another hallmark of progressive neurological dysfunction. Concurrently, neurobehavioral disorders such as anxiety, depression, and loss of motor skills may arise from the degeneration and loss of critical neuronal populations, including cholinergic, dopaminergic, and motor neurons, within the central nervous system (14). Various therapeutic strategies have therefore been developed to address these symptoms, with the aim of slowing disease progression and improving patient quality of life.

Recent advances in neuropharmacology have highlighted the substantial potential of flavonoids, a diverse group of plant-derived polyphenols, in modulating neurological health. These compounds, particularly apigenin, a flavone abundant in fruits, teas, and vegetables, have demonstrated neuroprotective, antioxidant, anti-inflammatory, antitumor, and antimicrobial properties in numerous experimental studies (15). Notably, apigenin readily crosses the blood-brain barrier, enabling direct effects on the central nervous system (16). Increasing evidence suggests that apigenin exerts antidepressant effects through its influence on α-adrenergic, dopaminergic, and serotonergic receptor systems (16). Its administration has been shown to restore monoamine neurotransmitter levels, such as serotonin, dopamine, and epinephrine, which are frequently dysregulated in depressive states (17). Furthermore, apigenin modulates critical intracellular signaling pathways, including the cAMP-CREB-BDNF axis and NMDA receptor-mediated processes, which are essential for neuronal survival, synaptic plasticity, cognition, and mood (17). Comprehensive pharmacological evaluations and literature reviews have consistently supported the potential of apigenin in mitigating neurological disorders.

The search for effective and well-tolerated interventions for depression and anxiety extends beyond conventional pharmacology. Recent systematic reviews have highlighted the potential of various non-pharmacological modalities, such as mindfulness-based therapy for symptoms in irritable bowel syndrome and pulsed electromagnetic field therapy for multiple sclerosis-related fatigue and depression, underscoring the importance of targeting neuropsychiatric symptoms in comorbid conditions and the value of multimodal approaches (18, 19). In this context, natural products with pleiotropic mechanisms of action and high safety margins, such as the flavonoid apigenin, represent a compelling avenue for investigation. Apigenin’s ability to concurrently modulate oxidative stress, neuroinflammation, neurotransmitter systems, and neurotrophic signaling mirrors the multitarget therapeutic strategy increasingly sought in neuropsychiatry.

Given the growing body of evidence supporting the neuroprotective properties of apigenin, together with the well-documented roles of neuroinflammation and oxidative stress in the development of depression and anxiety, this systematic review aimed to rigorously examine preclinical investigations of the anxiolytic and antidepressant effects of apigenin in rodent models.

## 2. Evidence Acquisition

### 2.1. Search Strategy

This systematic review investigated the antidepressant and anxiolytic efficacy of apigenin in rodent models in accordance with the Cochrane Handbook and PRISMA guidelines (20, 21). The review protocol was not prospectively registered in a public repository. Although the review question, eligibility criteria, search strategy, and data extraction framework were specified a priori and applied consistently throughout the review process, the absence of prospective registration should be considered a methodological limitation.

A comprehensive search was conducted independently by two investigators in May 2025 across MEDLINE/PubMed, Scopus, and Embase, covering the literature up to 24 May 2025, and was subsequently updated on 14 July 2025. The full electronic search strategies for PubMed, Scopus, and Embase, including database-specific syntax and the number of records retrieved from each source, are provided in Supplementary Appendix A (Figure S1). Gray literature was additionally searched using Google Scholar (first 20 pages, sorted by relevance). No restrictions were imposed by geography, study design, or language; non-English articles were translated using Google Translate. To ensure a comprehensive search, the reference lists of relevant studies and reviews were also screened. EndNote X9 was used for reference management and duplicate removal. The remaining articles underwent independent screening by the investigators at the title, abstract, and full-text levels to determine eligibility based on predefined inclusion criteria.

### 2.2. Selection Criteria

This review included peer-reviewed, preclinical in vivo studies evaluating the effects of apigenin in animal models using established laboratory methodologies. No publication date limits were applied. Studies were excluded if they addressed psychological disorders other than depression and anxiety, used apigenin derivatives rather than apigenin itself, or were not original research (e.g., reviews or letters). Studies lacking full-text availability, using apigenin analogs, or failing to meet predetermined quality benchmarks were also excluded.

### 2.3. Data Extraction and Quality Evaluation

Data extraction was performed using a standardized form developed in Microsoft Excel, and discrepancies were resolved through discussion among the researchers. Extracted information included: 1) study characteristics, including authors, publication year, and country of origin; 2) subject details, specifying the animal model and the method used to induce depression or anxiety; 3) intervention parameters, including treatment type, dosage, route of administration, comparator drugs (if any), delivery system (e.g., nanostructure platform), and the specific compound used; and 4) outcome assessments, including the directionality of the primary outcome (positive, negative, or unclear), implicated mechanisms of action, associated signaling pathways, and any reported organ toxicities. The methodological quality of the included animal studies was evaluated using SYRCLE's Risk of Bias tool (22) to assess potential biases related to selection, performance, detection, attrition, reporting, and other factors. In addition, the ARRIVE Essential 10 checklist (23) was used to assess the completeness of reporting in the included in vivo studies. The checklist covered 10 domains: study design, sample size, inclusion and exclusion criteria, randomization, blinding, outcome measures, statistical methods, experimental animals, experimental procedures, and results. For descriptive purposes, studies reporting 7 - 10 items were classified as having higher reporting completeness, those reporting 4 - 6 items as having moderate reporting completeness, and those reporting 1 - 3 items as having low reporting completeness. Supplementary figures and tables related to this analysis are available in the Supplementary File. Because ARRIVE is a reporting guideline rather than a direct measure of internal validity, these categories were not interpreted as equivalent to methodological quality; risk of bias was assessed separately using SYRCLE's Risk of Bias tool.

## 3. Results

### 3.1. Study Selection

**Figure 1.**
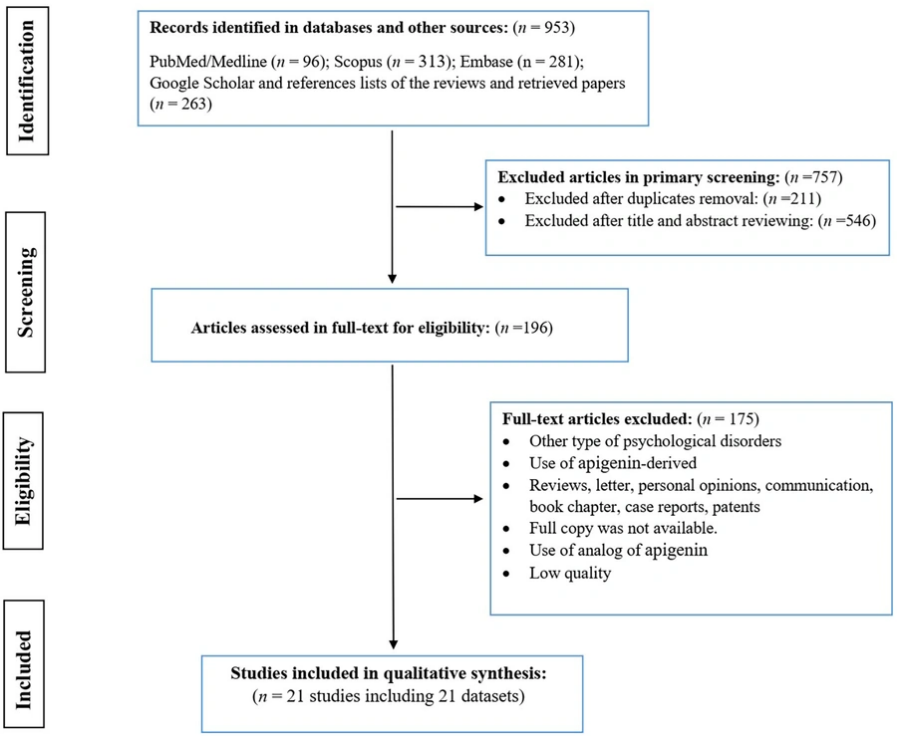
PRISMA flowchart for study selection

The systematic search identified 953 records from PubMed/MEDLINE (n = 96), Scopus (n = 313), Embase (n = 281), and supplementary sources, including Google Scholar and reference lists (n = 263). After removal of 211 duplicates, 742 records underwent title and abstract screening, and 546 records were excluded. The remaining 196 articles were assessed for full-text eligibility, of which 175 were excluded for reasons including a focus on disorders other than depression or anxiety, the use of apigenin derivatives, non-original research, a lack of full text, or low methodological quality. Ultimately, 21 studies met the inclusion criteria and were included in the qualitative synthesis (Figure 1).

### 3.2. General Characteristics of the Included Studies

The 21 included studies were published between 1998 and 2025, with 10/21 (47.62%) published between 2020 and 2025. Studies were conducted in 10 countries, with the largest contribution from China (8/21; 38.10%), followed by India (3/21; 14.30%) and Iran (2/21; 9.52%), with additional studies from Brazil, Iraq, Italy, Japan, Nigeria, Pakistan, and Saudi Arabia (Table 1). Rodent species included mice (12/21; 57.14%), rats (8/21; 38.10%), and both species (1/21; 4.76%) (Table 2). Of the 21 included studies, 14 (66.67%) were conducted in depression-only models, 5 (23.81%) in anxiety-only models, and 2 (9.52%) in combined depression/anxiety models (Table 1). These categories were used for descriptive classification and do not imply overlap in the reported totals. Detailed study-level characteristics and outcomes are summarized in Table 2.

**Table 1.** Characteristics of the Included Studies (N = 21)

Variables	No (%)
**Publication (y)**	
< 2000	1 (4.76)
2000 - 2009	4 (19.05)
2010 - 2019	6 (28.57)
2020 - 2025	10 (47.62)
Total	21 (100)
**Country**	
Brazil	1 (4.76)
China	8 (38.1)
India	3 (14.3)
Iran	2 (9.52)
Iraq	1 (4.76)
Italy	1 (4.76)
Japan	1 (4.76)
Nigeria	1 (4.76)
Pakistan	1 (4.76)
Saudi Arabia	2 (9.52)
Total	21 (100)
**Rodent**	
Mice	12 (57.14)
Mice and Rats	1 (4.76)
Rats	8 (38.1)
Total	21 (100)
**Disorder**	
Anxiety	5 (23.81)
Depression	14 (66.67)
Depression and Anxiety	2 (9.52)
Total	21 (100)

**Table 2.** Main Characteristics of Included Studies

Study	Induction	Treatment	Compare	Nano-Compound	Outcomes	Mechanism	Pathway	Toxicity
**Alghamdi et al. (2022) (25)**	21-day CMS	Apigenin 25/50 mg/kg p.o.	Imipramine 15 mg/kg p.o.	n/a	↑Locomotion, ↓corticosterone	Antioxidant, HPA normalization	↓Oxidative stress, ↑GSH	No toxicity
**Almzaien et al. (2022) (38)**	H_2_O_2_ in water (8 weeks)	Apigenin 150 mg/kg p.o.	BHT 25 mg/kg	n/a	↑Behavior, ↓MDA	Antioxidant, enzyme restoration	↓ROS, hippocampal protection	No toxicity
**Al-Yamani et al. (2022) (24)**	TST and MFST	Apigenin 25/50 mg/kg p.o.	Fluoxetine	n/a	↓Immobility, ↑activity	Monoamine-dependent	5-HT, DA, adrenergic	No toxicity
**Amin et al. (2022) (39)**	FST and EPM	Apigenin ± safranal	Fluoxetine	n/a	↓Immobility, ↓anxiety	5-HT1A/2A modulation	cAMP/PKA/CREB	No toxicity
**Bijani et al. (2022) (26)**	STZ i.c.v.	Apigenin 10 - 40 mg/kg i.p.	n/a	n/a	↑Behavior, ↓MDA	↓NLRP3/TLR4, ↑AMPK	Mitochondrial/inflammation	No toxicity
**Kumar and Bhat (2014) (40)**	EPM test	Apigenin 2.5 - 5 mg/kg p.o.	Diazepam	Apigenin 7-glucoside	Anxiolytic, diazepam-like	GABAA-BDZ modulation	↑GABAergic inhibition	No toxicity
**Kumar and Sharma (2006) (41)**	EPM test	Apigenin 0.5 - 5 mg/kg p.o.	Diazepam	n/a	Anxiolytic, dose-dependent	BDZ binding	GABAA receptor	No toxicity
**Li et al. (2015) (28)**	LPS i.p.	Apigenin 25 - 50 mg/kg i.p.	Fluoxetine	n/a	↓Immobility, ↓IL-1β	↓NF-κB, ↓cytokines	Inflammation suppression	No toxicity
**Li et al. (2016) (27)**	CUMS (3 weeks)	Apigenin 20 mg/kg p.o.	GW9662	n/a	↑Activity, ↓IL-1β/18	PPARγ activation	↓NLRP3 inflammasome	No toxicity
**Liu et al. (2020) (29)**	Corticosterone s.c.	Apigenin 20 mg/kg	Chrysanthemum, naringenin	n/a	↑Sucrose, ↓CORT	Metabolic correction	TCA, 5-HT, redox	No toxicity
**Mohammadkhanizadeh et al. (2024) (42)**	Social isolation	Apigenin 50 mg/kg p.o.	n/a	n/a	↑Memory, ↓anxiety	↓IL-6/MDA, ↑SOD/BDNF	Neuroplasticity via BDNF	No toxicity
**Nakazawa et al. (2003) (30)**	FST	Apigenin 12.5 - 100 mg/kg i.p.	Haloperidol	n/a	↓Immobility, ↑DA turnover	D2 modulation	Amygdala/hypothalamus DA	No toxicity
**Olayinka et al. (2023) (31)**	CUMS (14 days)	Apigenin 12.5 - 25 mg/kg i.p.	n/a	n/a	↑Mood, ↓MDA, ↑SOD	MAOA inhibition	Monoamine + redox	No toxicity
**Salgueiro et al. (1997) (43)**	EPM	Apigenin 10 mg/kg i.p.	Diazepam	n/a	Anxiolysis via BDZ	BDZ partial agonism	GABA modulation	No toxicity
**Sharma et al. (2018) (32)**	PTZ kindling	Apigenin 10 - 20 mg/kg p.o.	n/a	n/a	↑BDNF/CREB, ↓immobility	CREB-BDNF upregulation	Neuroplasticity	No toxicity
**Weng et al. (2016) (33)**	Corticosterone	Apigenin 20 - 40 mg/kg p.o.	Fluoxetine	n/a	↑BDNF, ↓anhedonia	BDNF-TrkB activation	HPA suppression	No toxicity
**Xie et al. (2025) (34)**	LiCl-pilocarpine	Apigenin 50 - 100 mg/kg p.o.	Valproate	n/a	↓Seizures, ↑neurogenesis	↑PI3K/AKT, ↓GFAP	Astrocyte suppression	No toxicity
**Yi et al. (2008) (35)**	FST and CMS	Apigenin 7 - 20 mg/kg p.o.	Fluoxetine	n/a	↑5-HT/DA, ↓CORT	↑Adenylyl cyclase	Monoamine + HPA	No toxicity
**Zanoli et al. (2000) (44)**	EPM, OFT	Apigenin 12.5 - 100 mg/kg i.p.	Diazepam	n/a	↓Locomotion, no anxiolysis	GABA-independent	Unknown	No toxicity
**Zhang et al. (2019) (36)**	Chronic restraint	Apigenin 20 - 60 mg/kg	Fluoxetine	n/a	↑Autophagy markers	↑AMPK, ↓mTOR	Autophagy + neuroplasticity	Mild in vitro toxicity
**Zhang et al. (2023) (37)**	Corticosterone s.c.	Apigenin 10 - 40 mg/kg p.o.	Fluoxetine	n/a	↑BDNF, ↓apoptosis	↑cAMP-CREB-BDNF	Neuroprotection, ↓ROS	No toxicity

Abbreviations: CMS, chronic mild stress; CUMS, chronic unpredictable mild stress; FST, forced swim test; TST, tail suspension test; MFST, modified forced swim test; EPM, elevated plus maze; SPT, sucrose preference test; OFT, open field test; MWM, Morris water maze; PCPA, para-chlorophenylalanine; AMPT, α-methyl-para-tyrosine; SCH, SCH-23390; i.p., intraperitoneal; p.o., per os/oral administration; s.c., subcutaneous; i.c.v., intracerebroventricular; BDNF, brain-derived neurotrophic factor; CREB, cAMP response element-binding protein; pCREB, phosphorylated CREB; cAMP, cyclic adenosine monophosphate; PKA, protein kinase A; GSH, glutathione; MDA, malondialdehyde; FRAP, ferric reducing antioxidant power; CoQ10, coenzyme Q10; ROS, reactive oxygen species; RNS, reactive nitrogen species; HPA axis, hypothalamic-pituitary-adrenal axis; MAOA, monoamine oxidase A; TNF-α, tumor necrosis factor alpha; IL-1β, interleukin-1 beta; IL-6, interleukin-6; NF-κB, nuclear factor kappa B; PPARγ, peroxisome proliferator-activated receptor gamma; NLRP3, NOD-, LRR-, and pyrin domain-containing protein 3; ASC, apoptosis-associated speck-like protein containing a CARD; GFAP, glial fibrillary acidic protein; DA, dopamine; 5-HT, 5-hydroxytryptamine/serotonin; NE, norepinephrine; BDZ, benzodiazepine; AKT, protein kinase B; PI3K, phosphoinositide 3-kinase; ULK1, Unc-51-like autophagy-activating kinase 1; mTOR, mechanistic target of rapamycin; Bax, Bcl-2-associated X protein; Bcl-2, B-cell lymphoma 2.

Depression-like and/or anxiety-like behaviors were assessed using validated behavioral paradigms. As summarized in Table 2, the most frequently used tests included the forced swim test (FST), tail suspension test (TST), open field test (OFT), elevated plus maze (EPM), the light-dark box, and sucrose preference test (SPT). Apigenin was administered orally or intraperitoneally at doses ranging from 1 to 150 mg/kg. Treatment duration varied across studies and included single-dose administration and repeated dosing protocols lasting from several days to multiple weeks. Control groups typically received vehicle treatment, and some studies included reference drugs such as fluoxetine or diazepam. The direction of behavioral effects (positive, negative, or unclear) and the specific tests employed are presented on a study-by-study basis in Table 2.

### 3.3. Risk of Bias and Reporting Quality

Risk of bias was assessed for all 21 included in vivo studies using SYRCLE's Risk of Bias tool across 10 domains (Figure S2 and Table S1 in the Supplementary File). Most studies were rated as having low risk for baseline characteristics, allocation concealment, selective outcome reporting, and other sources of bias (21/21; 100% for each domain). Low-risk ratings were also common for random housing (18/21; 85.71%) and incomplete outcome data (18/21; 85.71%). For sequence generation, 13/21 studies (61.90%) were rated as low risk, and 8/21 (38.10%) were rated as unclear/not evaluated. The domains most frequently rated as unclear/not evaluated were blinding (performance bias) (18/21; 85.71% unclear/not evaluated), random outcome assessment (19/21; 90.48% unclear/not evaluated), and blinding (detection bias) (19/21; 90.48% unclear/not evaluated). No domains were rated as high risk (Table S1).

Reporting completeness was evaluated using the ARRIVE Essential 10 checklist (Table S2 in the Supplementary File). Items consistently reported across studies included study design, sample size, outcome measures, statistical methods, experimental animals, experimental procedures, and results (21/21; 100% for each domain). In contrast, reporting was less consistent for inclusion and exclusion criteria (9/21; 42.86% reported; 12/21; 57.14% unclear/not evaluated), randomization (3/21; 14.29% reported; 18/21; 85.71% unclear/not evaluated), and blinding (6/21; 28.57% reported; 15/21; 71.43% unclear/not evaluated). Based on the predefined ARRIVE scoring threshold, all studies were classified as having higher overall reporting completeness in Table S2 in the Supplementary File, reflecting total scores of at least 7/10.

### 3.4. Behavioral Outcomes

Detailed behavioral outcomes and dosing regimens are presented in Table 2. Depression-like behaviors were evaluated in 16 of the included studies (14 depression-only and 2 depression/anxiety studies) (24, 25, 26, 27, 28, 29, 30, 31, 32, 33, 34, 35, 36, 37, 38, 39). In the forced swim test and tail suspension test, multiple studies reported reduced immobility time in apigenin-treated animals compared with control groups. In studies using the sucrose preference test, apigenin administration was associated with higher sucrose consumption relative to controls. Additional behavioral assessments included novelty-suppressed feeding and chronic mild stress paradigms. In these models, apigenin-treated animals exhibited differences in feeding latency or behavioral scores compared with untreated stressed animals, as reported by the respective studies (Table 2).

Anxiety-like behaviors were assessed in 7 studies (5 anxiety-only and 2 depression/anxiety studies) using tests such as the elevated plus maze, open field test, light-dark box, and marble burying test (38, 39, 40, 41, 42, 43, 44). Across these studies, apigenin-treated animals demonstrated changes in time spent in open arms, central zones, or illuminated compartments compared with control animals, depending on the specific behavioral paradigm employed. In the open field test, several studies reported increased central zone exploration or total distance traveled following apigenin administration. In the elevated plus maze, changes in open-arm entries and time spent in open arms were reported. Outcomes varied across studies by species, dose, route of administration, and experimental model (Table 2).

### 3.5. Mechanistic Insights

**Figure 2.**
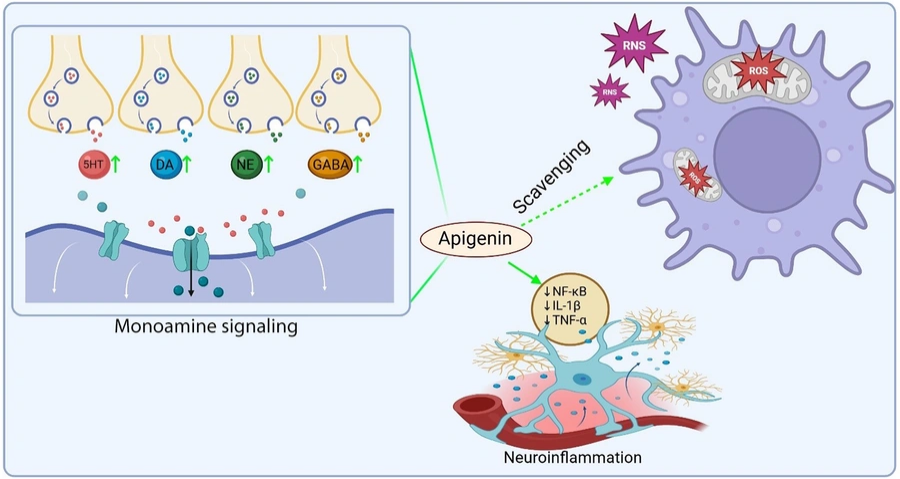
Mechanisms underlying the neuropsychological effects of apigenin. This figure depicts the modulation by apigenin of monoamine signaling through increased synaptic concentrations of 5-HT, DA, NE, and GABA, resulting in enhanced neurotransmission. The illustration also highlights the neuroprotective actions of apigenin, including scavenging of ROS and reactive nitrogen species (RNS), thereby reducing oxidative stress. Furthermore, the figure shows the attenuation by apigenin of neuroinflammation through the downregulation of pro-inflammatory mediators, such as NF-κB, IL-1β, and TNF-α. 5-HT, serotonin; DA, dopamine; NE, norepinephrine; GABA, gamma-aminobutyric acid; ROS, reactive oxygen species; RNS, reactive nitrogen species; NF-κB, nuclear factor kappa B; IL-1β, interleukin-1 beta; TNF-α, tumor necrosis factor-alpha.

Mechanistic endpoints were reported across multiple domains in the included studies (Table 2). Oxidative/antioxidant measures were reported in 7/21 studies (25, 26, 29, 31, 37, 38, 42), including lipid peroxidation and/or oxidative stress indices, such as MDA/ROS-related readouts, and antioxidant defenses, such as GSH and/or antioxidant enzymes. Inflammation-related outcomes were reported in 4/21 studies (26, 27, 28, 42), including cytokine-linked readouts, such as IL-1β/IL-6, and pathway-linked measures, with 1 study reporting NLRP3/TLR4-related targets (26). HPA axis-related measures (corticosterone-related readouts) were reported in 4/21 studies (25, 29, 31, 35). Monoaminergic/receptor-related outcomes were reported in 6/21 studies (24, 29, 30, 31, 35, 39), including serotonin/dopamine-related measures and/or metabolism/receptor-linked outcomes, such as MAOA- or 5-HT receptor-linked readouts. GABAergic/benzodiazepine-site-related outcomes were reported in 3/21 studies (40, 41, 43). In contrast, 1 study reported no anxiolytic effect and described the behavioral findings as GABA-independent, with an unknown pathway assignment (44). Neurotrophic/neuroplasticity-related outcomes were reported in 5/21 studies (32, 33, 37, 39, 42), including BDNF- and/or CREB-linked measures and BDNF-TrkB-linked readouts. Intracellular signaling outcomes were reported in 4/21 studies (26, 27, 34, 36), including AMPK/mTOR-related measures, PI3K/AKT-associated readouts with astrocyte markers, such as GFAP, and PPARγ-linked inflammasome-related outcomes. The specific markers measured and the direction of reported changes for each study are summarized in Table 2 and grouped by mechanistic domain in Figure 2.

### 3.6. Safety and Toxicity

Across all 21 studies, no overt toxicity or adverse behavioral effects were reported after apigenin administration, even at doses up to 150 mg/kg orally or 100 mg/kg intraperitoneally (Table 2). One study (36) noted mild in vitro toxicity at higher concentrations, but no in vivo toxicity was observed in animal models.

## 4. Conclusions

This systematic review synthesized preclinical evidence from 21 rodent studies evaluating the effects of apigenin on depression and anxiety. Overall, the findings suggest that apigenin exerts antidepressant- and anxiolytic-like effects across diverse behavioral paradigms, although the evidence base remains heterogeneous in terms of animal models, dosing regimens, routes of administration, and molecular endpoints.

The observed reductions in depressive-like behaviors, including immobility in the FST/TST and anhedonia in CMS, align with the pharmacological effects of conventional antidepressants such as fluoxetine and imipramine, as reported in several included studies (24, 25). Apigenin also showed anxiolytic-like effects in the EPM that were comparable to diazepam, but without the sedative effects typically associated with benzodiazepines (41). In addition, 1 included study reported synergistic behavioral improvement following the coadministration of apigenin and safranal (39), suggesting that combination approaches may warrant further investigation.

Our findings are consistent with prior reviews on flavonoids in neuropsychiatric disorders. For example, Olasehinde and Olaokun (17) highlighted the cognitive and neurobehavioral benefits of apigenin, while Popović et al. (16) noted its effects on memory retention. However, the present review extends these observations by systematically linking behavioral outcomes to specific molecular pathways, including AMPK/mTOR-mediated autophagy, PI3K/AKT-driven neurogenesis, and PPARγ-dependent anti-inflammatory effects. Unlike selective serotonin reuptake inhibitors, which primarily modulate monoamine levels, apigenin concurrently addresses oxidative stress, inflammation, and neurotrophic support, potentially resulting in broader efficacy and a faster onset of action, as suggested in some preclinical models (35, 36).

Based on the findings of this review, the included studies suggest that apigenin may act through several interconnected mechanisms, including the regulation of oxidative stress and neuroinflammation, monoaminergic and GABAergic neurotransmission, and neurotrophic and intracellular survival signaling pathways (Figure 2). This mechanistic profile is consistent with current views of depression and anxiety as multifactorial disorders involving oxidative imbalance, inflammatory processes, and impaired neurotrophic support. For example, reductions in oxidative markers such as MDA and nitrite, together with restoration of antioxidant defenses including GSH and catalase, align with previous evidence supporting the relevance of redox dysregulation in mood disorders and the antioxidant-related biological activity of apigenin (15, 37, 45, 46). Likewise, the reported suppression of TNF-α, IL-1β, and IL-6 and the inhibition of NF-κB- and NLRP3-related pathways support the growing recognition of neuroimmune involvement in depression and anxiety (7, 27, 45). Modulation of monoaminergic systems, particularly serotonergic and dopaminergic pathways, positions apigenin within the framework of classic antidepressant action, but with a broader mechanism encompassing receptor-level interactions, such as 5-HT1A agonism and 5-HT2A antagonism, and enzyme inhibition (MAOA) (24, 39). This is further supported by apigenin’s ability to enhance neurotrophic signaling through the CREB-BDNF axis, a pathway critically implicated in neuronal plasticity, resilience, and antidepressant efficacy (32, 33).

Despite promising preclinical outcomes, several critical translational gaps remain. First, pharmacokinetic data for apigenin in rodents are limited and suggest variable bioavailability depending on formulation and route of administration. Although apigenin is known to cross the blood-brain barrier (16), its half-life, tissue distribution, and metabolism in the central nervous system are not well characterized in the included studies. In humans, apigenin undergoes extensive phase II metabolism, including glucuronidation and sulfation, which may limit its systemic bioavailability and brain penetration when administered orally (47). Formulation strategies such as nanoencapsulation, lipid-based delivery, or prodrug approaches may be necessary to enhance its pharmacokinetic profile. Second, long-term toxicity data are notably absent from the reviewed studies, which predominantly assessed acute or subchronic dosing. Chronic exposure studies are essential to rule out potential organ toxicity, endocrine disruption, or off-target effects, especially given the pharmacological activity of apigenin on multiple receptor systems, such as GABAA, 5-HT, and adrenergic receptors. Furthermore, the safety of apigenin in vulnerable populations, such as those with hepatic or renal impairment or during pregnancy, remains unexplored. Third, the human relevance of rodent-based behavioral models is inherently limited. Although models such as CMS, FST, and EPM are well validated for screening antidepressant and anxiolytic agents, they do not fully capture the complexity of human affective disorders, which involve cognitive, social, and environmental dimensions that cannot be replicated in animals. In addition, the doses used in preclinical studies, often 10 - 150 mg/kg, may not translate directly to safe and effective human equivalents, necessitating careful dose-finding studies in early-phase clinical trials.

A key strength of this review is its synthesis of both behavioral and mechanistic evidence across preclinical studies, which revealed convergence on several relevant biological pathways despite variability in models and dosing. However, several limitations must be acknowledged. Exclusive reliance on rodent models precludes direct extrapolation to humans. Variability in apigenin doses, administration routes, and treatment durations complicates the establishment of optimal dosing regimens. The predominance of male rodents in the included studies limits understanding of potential sex-specific effects, which are increasingly recognized in mood disorder research. In addition, the general lack of long-term safety and pharmacokinetic data in vivo underscores the need for further preclinical profiling. The review protocol was not prospectively registered, which may reduce transparency and limit the ability to verify whether any methodological deviations occurred during the review process. Finally, although all studies met the predefined threshold for higher overall reporting completeness on the ARRIVE Essential 10 checklist, this mainly reflected consistent reporting of general study descriptors and outcomes. In contrast, several items with direct relevance to internal validity, particularly randomization, blinding, and inclusion/exclusion criteria, were frequently unclear or not reported. Therefore, the ARRIVE-based classification should be interpreted cautiously and should not be considered a substitute for risk-of-bias assessment. Future research should prioritize: 1) pharmacokinetic and bioavailability studies in relevant animal models and humans; 2) long-term toxicity and safety assessments, including in sensitive populations; 3) investigation of sex differences in the efficacy and safety of apigenin; and 4) clinical trials to evaluate the efficacy, safety, and optimal dosing of apigenin in patients with depression and anxiety disorders, particularly in treatment-resistant or comorbid populations.

In summary, preclinical studies suggest that apigenin has antidepressant- and anxiolytic-like effects in rodent models, supported by antioxidant, anti-inflammatory, monoaminergic, and neurotrophic mechanisms. Although these findings support further translational investigation, clinical relevance remains uncertain because the current evidence is limited to preclinical studies.

ijpr-25-1-167153-s001.pdf

## Data Availability

The data presented in this study are uploaded during submission as a supplementary file and are openly available for readers upon request.
