# Supplementary material for: Apigenin for Depression and Anxiety: A Systematic Review of Preclinical Studies
Source: Iran J Pharm Res. 2026 May 19;25(1):e167153. doi: 10.5812/ijpr-167153 (PMC13389377; doi:10.5812/ijpr-167153)
Supplement: ijpr-25-1-167153-s001.pdf [file ijpr-25-1-167153-s001.pdf]

PubMed
("apigenin"[Supplementary Concept] OR "apigenin"[All Fields] OR "apigenin"[MeSH Terms] OR "apigenin s"[All Fields] OR "apigenine"[All Fields]) AND (("depressed"[All Fields] OR "depression"[MeSH Terms] OR "depression"[All Fields] OR "depressions"[All Fields] OR "depression s"[All Fields] OR "depressive disorder"[MeSH Terms] OR ("depressive"[All Fields] AND "disorder"[All Fields]) OR "depressive disorder"[All Fields] OR "depressivity"[All Fields] OR "depressive"[All Fields] OR "depressively"[All Fields] OR "depressiveness"[All Fields] OR "depressives"[All Fields]) OR ("anxiety"[MeSH Terms] OR "anxiety"[All Fields] OR "anxieties"[All Fields] OR "anxiety s"[All Fields]))
Records retrieved: 96

Scopus
TITLE-ABS-KEY ((apigenin OR apigenine) AND (("depressed" OR "depression" OR "depressions" OR "depressivity" OR "depressive" OR "depressively" OR "depressiveness" OR "depressives" OR "depressive disorder") OR ("anxiety" OR "anxieties")))
Records retrieved: 313

Embase
('apigenin'/exp OR apigenin) AND ('depression'/exp OR depression OR 'anxiety'/exp OR anxiety)
Records retrieved: 281

Figure S1. Full electronic search strategies for PubMed, Scopus, and Embase, including database-specific syntax and number of records retrieved.

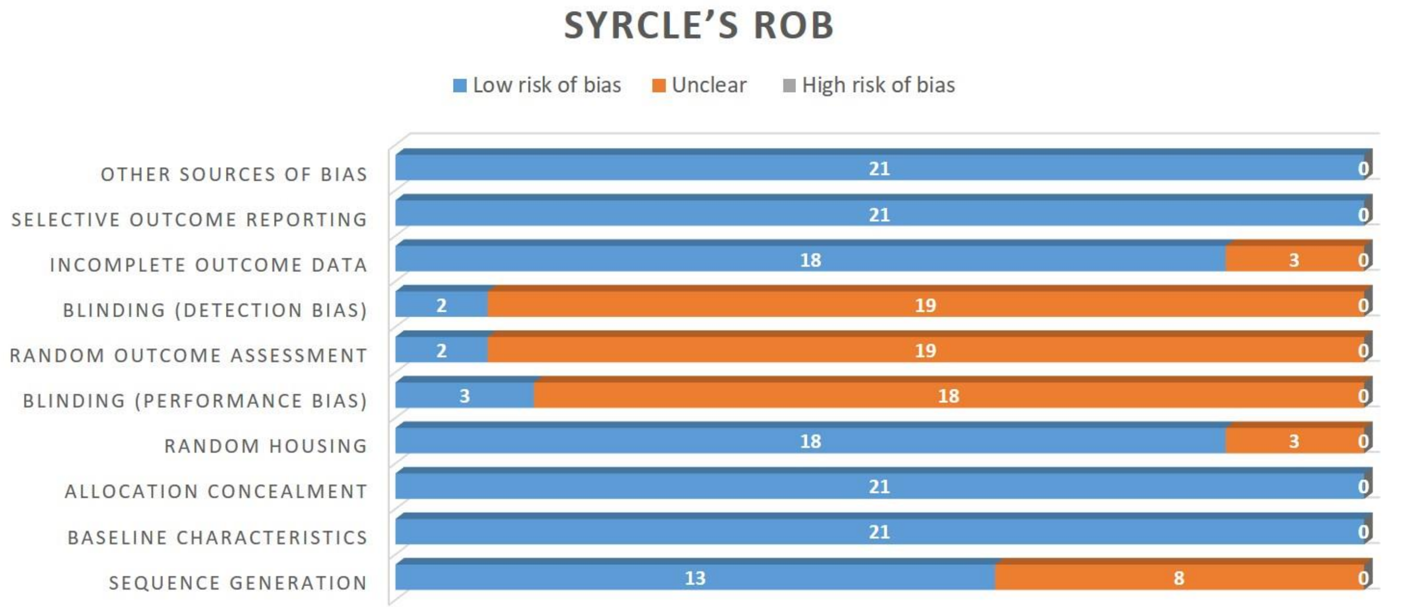

	Sequence generation	Baseline characteristics	Allocation concealment	Random housing	Blinding (Performance bias)	Random outcome assessment	Blinding (Detection bias)	Incomplete outcome data	Selective outcome reporting	Other sources of bias
Low risk of bias	13	21	21	18	3	2	2	18	21	21
Unclear	8	0	0	3	18	19	19	3	0	0
High risk of bias	0	0	0	0	0	0	0	0	0	0

Figure S2. SYRCLE's Risk-of-Bias Assessment Across Ten Methodological Domains in 21 Preclinical Studies.

Table S1. Summary of Risk of Bias Evaluation for in Vivo Studies Using SYRCLE's RoB Instrument

**Study**	**Sequence generation**	**Baseline characteristics**	**Allocation concealment**	**Random housing**	**Blinding (Performance bias)**	**Random outcome assessment**	**Blinding (Detection bias)**	**Incomplete outcome data**	**Selective outcome reporting**	**Other sources of bias**
Alghamdi et al. (2022)	y	y	y	y	Uc/NE	Uc/NE	uc/NE	y	y	y
Almzaien et al. (2022)	Uc/NE	y	y	Uc/NE	Uc/NE	Uc/NE	y	uc/NE	y	y
Al-Yamani et al. (2022)	Uc/NE	y	y	y	y	Uc/NE	uc/NE	y	y	y
Amin et al. (2022)	y	y	y	y	Uc/NE	Uc/NE	uc/NE	y	y	y
Bijani et al. (2022).	y	y	y	y	Uc/NE	Uc/NE	uc/NE	y	y	y
Kumar & Bhat (2014)	Uc/NE	y	y	y	Uc/NE	y	uc/NE	y	y	y
Kumar & Sharma (2006)	Uc/NE	y	y	y	Uc/NE	Uc/NE	uc/NE	uc/NE	y	y
Li et al. (2015)	y	y	y	Uc/NE	Uc/NE	Uc/NE	uc/NE	y	y	y
Li et al. (2016)	Uc/NE	y	y	y	Uc/NE	Uc/NE	uc/NE	y	y	y
Liu T. et al. (2020)	y	y	y	Uc/NE	y	Uc/NE	uc/NE	y	y	y
Mohammadkhanizadeh et al. (2024).	Uc/NE	y	y	y	Uc/NE	Uc/NE	y	y	y	y
Nakazawa et al. (2003)	y	y	y	y	Uc/NE	Uc/NE	uc/NE	y	y	y
Olayinka et al. (2023)	Uc/NE	y	y	y	Uc/NE	Uc/NE	uc/NE	uc/NE	y	y
Salgueiro et al. (1997)	y	y	y	y	y	Uc/NE	uc/NE	y	y	y
Sharma et al. (2018)	y	y	y	y	Uc/NE	Uc/NE	uc/NE	y	y	y
Weng et al. (2016).	y	y	y	y	Uc/NE	Uc/NE	uc/NE	y	y	y
Xie et al. (2025).	y	y	y	y	Uc/NE	Uc/NE	uc/NE	y	y	y
Yi et al. (2008)	y	y	y	y	Uc/NE	Uc/NE	uc/NE	y	y	y
Zanoli et al. (2000)	Uc/NE	y	y	y	Uc/NE	y	uc/NE	y	y	y
Zhang et al. (2019).	y	y	y	y	Uc/NE	Uc/NE	uc/NE	y	y	y
Zhang et al. (2023).	y	y	y	y	Uc/NE	Uc/NE	uc/NE	y	y	y

y, low risk of bias; X, high risk of bias; Uc/NE, unclear/not evaluated

Table S2. Overview of Reporting Completeness for In Vivo Studies Based on the ARRIVE Essential 10 Checklist

**Study**	1	2	3	4	5	6	7	8	9	10	Reporting completeness
Alghamdi et al. (2022)	y	y	Uc/NE	Uc/NE	Uc/NE	y	y	y	y	y	Higher reporting completeness
Almzaien et al. (2022)	y	y	Uc/NE	y	Uc/NE	y	y	y	y	y	Higher reporting completeness
Al-Yamani et al. (2022)	y	y	Uc/NE	Uc/NE	y	y	y	y	y	y	Higher reporting completeness
Amin et al. (2022)	y	y	y	Uc/NE	Uc/NE	y	y	y	y	y	Higher reporting completeness
Bijani et al. (2022).	y	y	Uc/NE	Uc/NE	y	y	y	y	y	y	Higher reporting completeness
Kumar & Bhat (2014)	y	y	y	Uc/NE	Uc/NE	y	y	y	y	y	Higher reporting completeness
Kumar & Sharma (2006)	y	y	y	y	Uc/NE	y	y	y	y	y	Higher reporting completeness
Li et al. (2015)	y	y	Uc/NE	Uc/NE	y	y	y	y	y	y	Higher reporting completeness
Li et al. (2016)	y	y	Uc/NE	Uc/NE	Uc/NE	y	y	y	y	y	Higher reporting completeness
Liu T. et al. (2020)	y	y	y	Uc/NE	Uc/NE	y	y	y	y	y	Higher reporting completeness
Mohammadkhanizadeh et al. (2024).	y	y	Uc/NE	Uc/NE	Uc/NE	y	y	y	y	y	Higher reporting completeness
Nakazawa et al. (2003)	y	y	y	Uc/NE	Uc/NE	y	y	y	y	y	Higher reporting completeness
Olayinka et al. (2023)	y	y	Uc/NE	Uc/NE	y	y	y	y	y	y	Higher reporting completeness
Salgueiro et al. (1997)	y	y	Uc/NE	Uc/NE	Uc/NE	y	y	y	y	y	Higher reporting completeness
Sharma et al. (2018)	y	y	y	Uc/NE	Uc/NE	y	y	y	y	y	Higher reporting completeness
Weng et al. (2016).	y	y	y	Uc/NE	Uc/NE	y	y	y	y	y	Higher reporting completeness
Xie et al. (2025).	y	y	Uc/NE	Uc/NE	y	y	y	y	y	y	Higher reporting completeness
Yi et al. (2008)	y	y	y	Uc/NE	Uc/NE	y	y	y	y	y	Higher reporting completeness
Zanoli et al. (2000)	y	y	y	y	Uc/NE	y	y	y	y	y	Higher reporting completeness
Zhang et al. (2019).	y	y	Uc/NE	Uc/NE	y	y	y	y	y	y	Higher reporting completeness
Zhang et al. (2023).	y	y	Uc/NE	Uc/NE	Uc/NE	y	y	y	y	y	Higher reporting completeness

y, yes; X, no; Uc/NE, unclear/not evaluated

The ARRIVE Essential 10 checklist items: 1, Study Design; 2, Sample Size; 3, Inclusion and Exclusion Criteria; 4, Randomisation; 5, Blinding; 6, Outcome Measures; 7, Statistical Methods; 8, Experimental Animals; 9, Experimental Procedures; 10, Results.
